# Epidemiology, clinical characteristics, and outcome in candidemia: a retrospective five-year analysis from two tertiary general hospitals

**DOI:** 10.1186/s12879-025-10908-4

**Published:** 2025-04-11

**Authors:** Tingting Liu, Shuhong Sun, Xiaosong Zhu, Hui Wu, Zhiqing Sun, Shanxin Peng

**Affiliations:** 1 Department of Vasculocardiology, Linyi People’s Hospital, Shandong Second Medical University, Linyi, 276000 Shandong China; 2 Department of Clinical Microbiology, Linyi People’s Hospital, Shandong Second Medical University, Linyi, 276000 Shandong China; 3 Department of Infection Management, Linyi People’s Hospital, Shandong Second Medical University, Linyi, 276000 Shandong China; 4 Department of Clinical Microbiology, Linyi Central Hospital, Yishui, 276400 Shandong China

**Keywords:** Candidemia, *Candida* species, Antifungal susceptibility, Mortality, Risk factors

## Abstract

**Background:**

Candidemia is linked with high mortality, highlighting the critical importance of timely empirical antimicrobial therapy and precise medical intervention before a definite etiologic diagnosis. The current study aimed to investigate the prevalence of pathogens in patients with candidemia and evaluate the potential independent risk factors for *Candida albicans* bloodstream infections (BSI), as well as the prognosis of candidemia.

**Methods:**

A retrospective bicentric observational study was performed, incorporating 132 candidemia episodes from two tertiary general hospitals in the Linyi area between January 2019 and December 2023. Data on demographic characteristics, underlying diseases, medical intervention, and antimicrobial sensitivity were collected and analyzed using SPSS version 27.0. Univariate analysis and binary logistic regression analyses were performed to identify risk factors for non*-albicans Candida* infections and candidemia-related mortality.

**Results:**

A total of 132 strains of *Candida* species were isolated from 132 patients with candidemia, with non*-albicans Candida* accounting for 71.97% (95/132) and *Candida albicans* for 28.03%. Although *Candida albicans* remains the predominant species, the proportion of *Candida tropicalis*, mainly from the Hematology Ward, is approaching that of *Candida albicans*, which was mainly found in the intensive care unit (ICU) (27.27% versus 28.03%). Moreover, *Candida tropicalis*, the most frequently isolated non*-albicans Candida* species, exhibited poorer sensitivity to triazole drugs than other *Candida* species. Multivariate analysis identified gastrointestinal surgery (non-tumor) as an independent risk factor for *Candida albicans* BSI (odds ratio [OR] = 6.683, 95% confidence interval [CI]: 1.253–35.632, *P* = 0.026). The 30-day mortality rate of candidemia in the current study was 30.3%. Binary logistic regression analysis identified several factors significantly associated with mortality, including age (OR = 1.038, 95% CI: 1.007–1.071, *P* = 0.018) and septic shock (OR = 3.307, 95% CI: 1.205–9.071, *P* = 0.020).

**Conclusion:**

The mortality rate of candidemia in the current study reached 30.3%, indicating a high disease burden. Recently, the proportion of non*-albicans Candida*, especially *Candida tropicalis*, has increased markedly. Therefore, increased attention should be given to patients with the identified risk factors to improve candidemia management and outcomes.

**Clinical trial number:**

Not applicable.

## Introduction

Candidemia, the most common clinical form of invasive candidiasis, imposes a significant financial burden on the healthcare system [1, 2]. Epidemiology research indicates that its global incidence is increasing annually [3], making *Candida spp.* the fourth most common cause of nosocomial bloodstream infections (BSI) in the United States and the leading cause of fungal sepsis in Europe [4]. With a 30-day mortality rate of 37% following a positive blood culture, candidemia represents a serious public health threat [5].

An increased risk of candidemia has been closely linked to several factors, such as immunosuppression, excessive use of broad-spectrum antibiotics, and invasive interventions. Due to the slow growth rate of yeasts, the identification and antifungal susceptibility testing of *Candida* spp. take considerate time, complicating early diagnosis and targeted treatment [4]. However, early medical intervention, including effective antifungal therapy and supportive measures, is crucial for patient outcomes. Thus, early empirical antimicrobial therapy based on epidemiological data is necessary for improving the prognosis of patients with fungemia.

Due to the mild incipient symptoms of sepsis, non-special clinical signs, and the slow progression of candidemia, the condition is often masked by the primary disease or other associated infections. Moreover, the clinical distribution and characteristics of *Candida* spp. varies by region, hospitals, and even departments [6, 7]. The proportion of non-*albicans Candida* infection has increased significantly, with these species exhibiting higher resistance to traditional antifungal drugs [8]. As a result, investigating the epidemiology, drug resistance, and mortality risk factors of candidemia is crucial for identifying high-risk patients, enabling early treatment, and reducing mortality.

The current study aimed to examine strain distribution, drug susceptibility, and the clinical characteristics of patients with candidemia. We compared *Candida albicans* and non-*albicans Candida* species in terms of underlying conditions, potential risk factors, and clinical outcomes. Additionally, we sought to identify independent risk factors affecting prognosis, ultimately providing guidance for empirical drug selection and enhancing candidemia treatment efficacy.

## Materials and methods

### Study population

The current retrospective study was conducted in two tertiary general hospitals, both classified as Comprehensive Top-grade-A-level Hospitals, treating patients with tumors, hematological disease, trauma, and emergency conditions. Patients who met the diagnostic criteria [9] for candidemia from January 2019 to December 2023 were included. Candidemia was defined as at least one positive blood culture containing *Candida* species, accompanied by clinical symptoms and signs of *Candida* infection. Inclusion criteria included the following: (1) Meeting the aforementioned definition of candidemia; (2) being 18 years and older; and (3) having complete clinical data. Exclusion criteria included the following: (1) Prior prophylactic antifungal use before *Candida* detection and (2) mixed *Candida* species infections. The requirement for informed consent was waived due to the current study’s retrospective and observational design, and patient data were anonymized before analysis. The current study was conducted following the Declaration of Helsinki and was approved by the the Science and Technology Ethics Committee of Linyi People’s Hospital (approval No.202408-H-013).

### Data collection and definition

Data regarding demographic characteristics (gender and age), underlying diseases (including but not limited to malignancies, diabetes mellitus, and hypertension), infection severity(fever, concomitant infections, and septic shock), invasive operation (mechanical ventilation, central venous catheterization, and indwelling urinary catheterization), intensive care unit (ICU) admission, surgery within three months before fungemia, and outcome (30-day mortality) were collected from patients’ electronic medical records.

Neutropenia was defined as an absolute neutrophil count of < 500/mm^3^.

Based on 30-day mortality after disease onset (calculated from the time of the first positive blood culture), patients were divided into survivor and non-survivor groups. Non-survivors were those who died within 30 days of *Candida* detection in their blood culture.

### Species identification

Blood samples were collected under sterile conditions and deposited in a fully automated blood culture system (BACT/ALERT-3D, BioMérieux, France). Upon receiving a positive blood culture alert from the instrument, specimens from the positive blood bottles were first examined under a microscope and then inoculated onto 5% sheep blood agar and chocolate agar. Isolates were identified using matrix-assisted laser desorption ionization time-of-flight (MALDI/TOF, BioMérieux, France) analysis, previously described [10]. Briefly, a moderate number of colonies were directly applied to the target plate, followed by the addition of 0.5 µL of formic acid. After air drying, 1 µL of matrix solution, such as CHCA or HCCA, was added. Subsequently, yeast mass spectrometry data were collected using a machine, and the resulting yeast spectrum was compared with database spectra for comprehensive analysis. Before each analysis, the system was calibrated using the Escherichia coli DH5 strain. Only the initial episode of candidemia for each patient was considered.

### Antifungal susceptibility testing

Antifungal susceptibility testing (AFST) was performed using the ATB FUNGUS 3 yeast antifungal susceptibility test kit (BioMérieux, France), which included amphotericin B, fluconazole, itraconazole, and voriconazole. The tested antifungal concentrations ranged from 0.5 to 16 mg/L for amphotericin B, 1 to 128 mg/L for fluconazole, 0.125 to 4 mg/L for itraconazole, and 0.06 to 8 mg/L for voriconazole. After a 24-h incubation period, the minimum inhibitory concentration (MIC) was visually assessed, with amphotericin B exhibiting complete (100%) growth inhibition and azoles demonstrating 50% growth inhibition. The AFST results were interpreted according to CLSI M27-Ed4 and M59-E3. Briefly, *Candida albicans*, *Candida tropicalis*, and *Candida parapsilosis* isolates with an MIC ≥ 8 µg/mL and *Candida glabrata* isolates with an MIC ≥ 64 µg/mL were considered resistant to fluconazole. Isolates of *Candida albicans*,* Candida tropicalis* and *Candida parapsilosis* with an MIC ≥ 1 µg/mL were considered resistant to voriconazole [11]. Quality control strains used were *Candida albicans* (ATCC 90028) and *Candida krusei* (ATCC 6258).

### Statistical analysis

Data were analyzed using SPSS version 27.0. Categorical data are expressed as numbers (%), whereas continuous variables with a non-normal distribution are presented as medians (*interquartile range*). To compare differences between two patient groups (*Candida albicans* versus non-*albicans Candida* or good prognosis versus poor prognosis), the Chi-square test or Fisher’s exact test was used for categorical variables, and the nonparametric Mann–Whitney U test was used for continuous variables with non-normal distribution. Factors with a *P* value < 0.05 in the univariate analysis were incorporated into a binary logistic regression model to determine the independent risk factors. Statistical significance was defined as a two-tailed *P* < 0.05.

## Results

### Distribution of *Candida* species isolated from candidemia patients

From January 2019 to December 2023, 132 *Candida* strains were isolated from 132 patients with candidemia. The distribution of these *Candida* strains is presented in Table 1. *Candida albicans* was the most frequently isolated *Candida* species, accounting for 28.03% (37/132), whereas non-*albicans Candida* species comprised 71.97% (95/132). No cases of mixed infections involving two or more *Candida* species were identified.

The incidence of candidemia in both the ICU and Medical Wards (Hematology Ward and other Medical Wards) was comparable (37.88%, 50/132; 40.90%, 54/132, respectively) and was higher than in Surgical Wards (21.21% and 28/132). Among Medical Wards, the incidence of candidemia in the Hematology Ward was 40.74% (22/54). In the Hematology Ward, *Candida tropicalis* had the highest proportion, accounting for 86.36% (19/22).

**Fig. 1:**
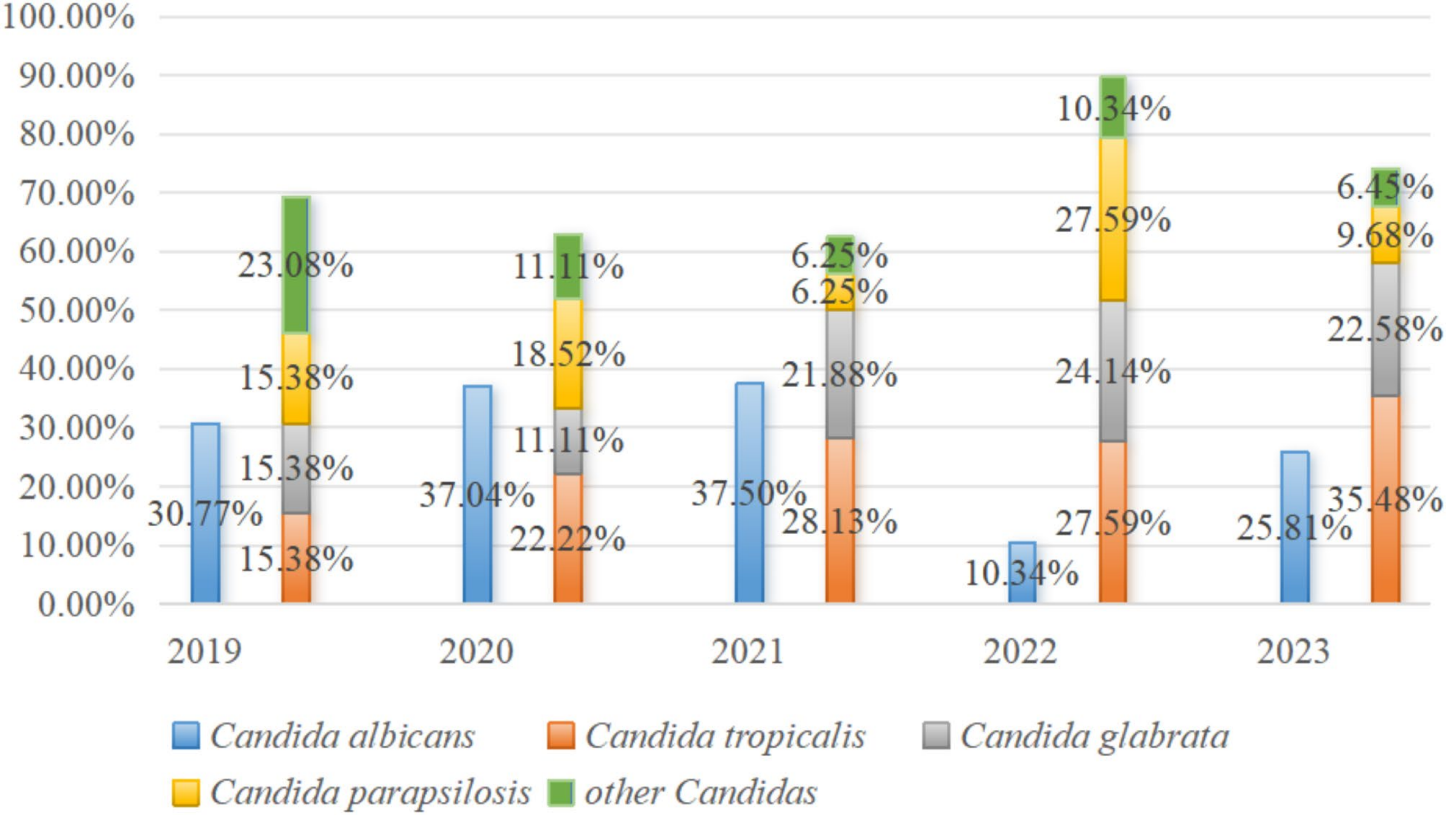
Annual composition of *Candida*

The annual composition of *Candida* species is illustrated in Fig. 1. The proportion of non-*albicans Candida*, especially *Candida tropicalis*, has increased over the last five years.

**Table 1:** Distribution of *Candida* species isolated from candidemia cases in clinical settings [n (%)]

Fungal species	Total	Intensive Care Unit	Hematology Ward	Other Medical Wards	Surgery Wards
*Candida albicans*	37(28.03)	19(38.00)	1(4.55)	8(25.00)	9(32.14)
*Candida tropicalis*	36(27.27)	12(24.00)	19(86.35)	4(12.50)	1(3.57)
*Candida glabrata*	26(19.70)	9(18.00)	0(0.00)	10(31.25)	7(25.00)
*Candida parapsilosis*	18(13.64)	6(12.00)	1(4.55)	3(9.38)	8(28.57)
Others*	15(11.36)	4(8.00)	1(4.55)	7(21.87)	3(10.72)
Total	132(100.00)	50(100.00)	22(100.00)	32(100.00)	28(100.00)

*Other *Candida* species included *Candida guilliermondii*,* Candida pelliculosa*,* Candida utilis*,* Candida krusei*,* Rhodotorulamucilaginosa*,* Candida lusitaniae*, and *Candida innominate.*

### Analysis of antifungal susceptibility in *Candida* species

Antimicrobial susceptibility testing revealed that *Candida albicans* was 91.00% sensitive to fluconazole and voriconazole. *Candida glabrata* exhibited higher sensitivity to voriconazole than to fluconazole and itraconazole. *Candida tropicalis* had sensitivity rates of 75.00% for both fluconazole and voriconazole and 61.11% for itraconazole. According to epidemiological cut-off values, all *Candida* species were 100% sensitive to amphotericin B (Table 2).

**Table 2:** Drug sensitivity analysis of major *Candida* strains in candidemia [n (%)]

Fungal species	*Candida albicans*		*Candida tropicalis*		*Candida glabrata*		*Candida parapsilosis*	
	S/WT	*R*	S/WT	*R*	S/WT	*R*	S/WT	*R*
Amphotericin B	37(100.00)	0	36(100.00)	0	26(100.00)	0	18(100.00)	0
Fluconazole	34(91.89)	0	27(75.00)	9(25.00)	24(92.31)	0	18(100.00)	0
Voriconazole	34(91.89)	1(2.70)	27(75.00)	6(16.67)	26(100.0)	0	18(100.00)	0
Itraconazole	-	-	22(61.11)	7(19.44)	23(88.46)	0	18(100.00)	0

S, susceptible; WT, wild type; R, resistant; - indicates the absence of clinical breakpoints or epidemiological cutoff values values

### Clinical characteristics of patients with candidemia

A total of 132 patients diagnosed with candidemia were enrolled in the current study. The median age was 60 years, and 62.12% (82/132) were male. Their demographics and clinical characteristics are presented in Table 3. The majority of patients (96.97%, 128/132) had underlying diseases at the time of diagnosis, with the most common comorbidities being non-tumor respiratory diseases (51.52%, 68/132) and concomitant bacteremia (31.82%, 42/132). Other predisposing factors for candidemia included fever (76.52%, 101/132), indwelling urinary catheters (63.64%, 84/132), central venous catheters (59.09%, 78/132), and ICU admission (50.00%, 66/132). Among the current study population, 40 patients died, yielding a mortality rate of 30.30%.

**Fig. 2:**
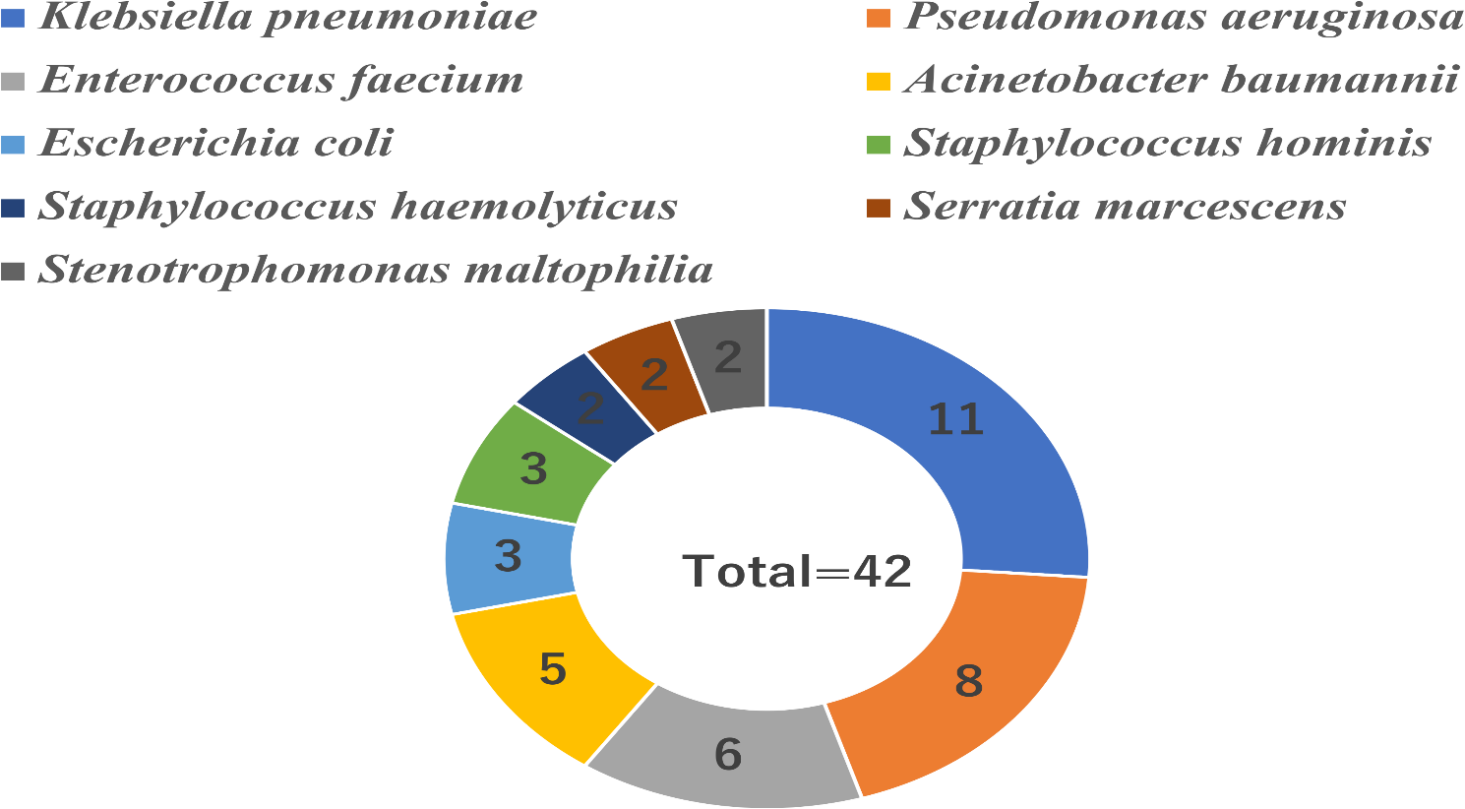
The constitution of bacteria isolated from patients with concomitant bacteremia

Furthermore, among 42 candidemia patients with concomitant bacteremia, nine bacterial species were isolated, totaling 42 bacteria strains. The most common species was *Klebsiella pneumoniae* (11/42, 26.19%), followed by *Pseudomonas aeruginosa* (8/42, 19.05%), *Enterococcus faecium* (6/42, 14.29%), *Acinetobacter baumannii* (5/42, 11.90%), *Escherichia coli* (3/42, 7.14%), *Staphylococcus hominis* (3/42, 7.14%), *Serratia marcescens* (2/42, 4.76%), *Stenotrophomonas maltophilia* (2/42, 4.76%), and *Staphylococcus haemolyticus* (2/42, 4.76%), as displayed in Fig. 2.

**Table 3:** Univariate analysis on factors related to BSI with *Candida albicans* and non-*albicans Candida*

Variable	Total (*n* = 132)	*Candida albicans* (*n* = 37)	non-*albicans Candida* (*n* = 95)	χ^2^/Z value	*P* value
Gender/Male	82(62.12)	21(56.76)	61(64.21)	0.629	0.428
Age/Years	60(50,70.75)	65(52,71)	59(50,70)	-1.305	0.192
Diabetes mellitus	38(28.79)	10(27.03)	28(29.47)	0.078	0.780
Hypertension	29(21.79)	6(16.22)	23(24.21)	0.993	0.319
Fever	101(76.52)	32(86.49)	69(72.63)	2.845	0.092
Concomitant bacteremia	42(31.82)	10(27.03)	32(33.68)	0.544	0.461
Hematological malignancy	22(16.67)	1(2.70)	21(22.11)	7.218	0.007
Solid malignancy	20(15.15)	4(10.81)	16(16.84)	0.753	0.385
Severe trauma	11(8.33)	3(8.11)	8(8.42)	0.003	0.953
Nervous system disease	39(29.55)	12(32.43)	27(28.42)	0.206	0.650
Respiratory Diseases (non-tumor)	68(51.52)	21(56.76)	47(49.47)	0.566	0.452
Intra-abdominal infections	0	0	0	N/A*	N/A
Intra-abdominal abscess	0	0	0	N/A	N/A
Gastrointestinal surgery (non-tumor)	8(6.06)	6(16.22)	2(2.11)	6.958	0.008
Gastrointestinal internal medicine diseases (non-tumor)	22(16.67)	8(21.62)	14(14.74)	2.190	0.139
Hepatobiliary and pancreatic diseases (non-tumor)	32(24.24)	10(27.03)	22(23.16)	0.217	0.641
Urinary system disease (non-tumor)	36(27.27)	10(27.03)	26(27.37)	0.002	0.968
Infective endocarditis	0	0	0	N/A	N/A
Neutropenia	0	0	0	N/A	N/A
Hemodialysis	0	0	0	N/A	N/A
Surgery	38(28.79)	14(37.84)	24(25.26)	2.054	0.152
Mechanical ventilation	54(40.91)	21(56.76)	33(34.74)	5.341	0.021
Central venous catheter	78(59.09)	23(62.16)	55(57.89)	0.201	0.654
Indwelling urinary catheter	84(63.64)	27(72.97)	57(60.00)	1.937	0.164
ICU admissions	66(50.00)	23(62.16)	43(45.26)	3.042	0.081
Poor prognosis	40(30.30)	10(27.03)	30(31.58)	0.261	0.609

Note: * Not applicable

### Risk factors for incidence of *Candida albicans* BSI

These patients were divided into two groups based on BSI with either *Candida albicans* (28.03%, 37/132) or non-*albicans Candida* (71.97%, 95/132). Univariate analysis revealed statistically significant differences between these groups regarding hematological malignancy, gastrointestinal surgery (non-tumor), and mechanical ventilation (*P* < 0.05, Table 3). However, there was no statistically significant difference between the two groups regarding age, gender, fever, concomitant bacteremia, other underlying diseases, central venous catheter, indwelling urinary catheter, surgery, ICU admissions, or poor prognosis (*P* ≥ 0.05, Table 3). Factors with *P* < 0.05 in the univariate analysis were included in the multivariate analysis, which revealed that gastrointestinal surgery (non-tumor) was an independent risk factor for *Candida albicans* BSI (odds ratio [OR] = 6.683, 95% confidence interval [CI]: 1.253–35.632, *P* = 0.026).

### Risk factors for mortality in patients with candidemia

Univariate analysis revealed that age, hypoproteinemia, septic shock, fever, respiratory diseases (non-tumor), central venous catheter use, catheter-related bloodstream infections or central line-associated bloodstream infections (CRBSI/CLABSI), and ICU admissions were associated with mortality in patients with candidemia (*P* < 0.05, Table 4). However, no statistically significant differences were observed between the two groups regarding gender, concomitant bacteremia, other underlying diseases, mechanical ventilation, indwelling urinary catheter, surgery, removal of the central venous catheter, therapeutic use of caspofungin, or *Candida albicans* (*P* ≥ 0.05, Table 4). After adjusting for confounding factors, multivariate logistic regression analysis identified age (OR = 1.038, 95% CI: 1.007–1.071, *P* = 0.018) and septic shock (OR = 3.307, 95% CI: 1.205–9.071, *P* = 0.020) as independent risk factors for mortality in patients with candidemia.

**Table 4:** Univariate analysis of the prognosis of patients with candidemia

Variable	Survivors (*n* = 92)	Non-Survivors (*n* = 40)	χ^2^/Z value	*P* value
Gender/Male	58(63.04)	24(60.00)	0.11	0.74
Age/Years	58(49,67)	69(56.5,76)	-3.557	0.000
Diabetes mellitus	27(29.35)	11(27.50)	0.046	0.829
Hypertension	19(20.65)	10(25.00)	0.307	0.579
Hypoproteinemia	9(9.78)	11(27.50)	6.807	0.009
Septic shock	15(16.30)	20(50.00)	16.245	0.000
Fever	65(70.65)	36(90.00)	5.808	0.016
Concomitant bacteremia	27(29.35)	15(37.50)	0.854	0.355
Hematological malignancy	14(15.22)	8(20.00)	0.459	0.498
Solid malignancy	17(18.48)	3(7.50)	2.614	0.106
Severe trauma	9(9.78)	2(5.00)	0.835	0.361
Nervous system disease	28(30.43)	11(27.50)	0.115	0.734
Respiratory diseases (non-tumor)	40(43.48)	28(70.00)	7.851	0.005
Gastrointestinal surgery (non-tumor)	6(6.52)	2(5.00)	0.113	0.736
Gastrointestinal internal medicine diseases (non-tumor)	15(16.30)	7(17.50)	0.029	0.865
Hepatobiliary and pancreatic diseases (non-tumor)	21(22.83)	11(27.50)	0.332	0.565
Urinary system disease (non-tumor)	23(25.00)	13(32.50)	0.791	0.374
Surgery	28(30.43)	10(25.00)	0.402	0.526
Mechanical ventilation	33(35.87)	21(52.50)	3.19	0.074
Central venous catheter	48(52.17)	30(75.00)	6.009	0.014
CRBSI/CLABSI	15(16.30)	13(32.50)	4.375	0.036
Removal of central venous catheter	14(15.22)	5(12.50)	0.167	0.683
Indwelling urinary catheter	56(60.87)	28(70.00)	1.004	0.316
ICU admissions	37(40.22)	29(72.50)	11.622	0.001
Therapeutic use of caspofungin	19(20.65)	13(32.50)	2.131	0.144
*Candida albicans*	27(29.35)	10(25.00)	0.261	0.609

## Discussion

Candidemia is a significant public health concern, characterized by high mortality rates and substantial healthcare expenditures. Understanding regional epidemiological trends and antifungal resistance patterns is imperative for clinicians to formulate evidence-based prophylactic treatments, especially during the interval before culture-dependent antimicrobial susceptibility data become available [12]. Given the significant geographical variations in *Candida* strain distribution [12, 13], we conducted a five-year retrospective surveillance study on candidemia in the Linyi region.

In the current study, *Candida albicans* was the most frequently isolated species (28.03%), followed by *Candida tropicalis* (27.27%), *Candida glabrata* (19.70%), *Candida parapsilosis* (13.64%), and other *Candida* species (11.36%). Compared to a study conducted in Shanghai (2008–2012) [14], we observed an increased detection of *Candida tropicalis* and *Candida glabrata*, along with a decline in *Candida albicans* and *Candida parapsilosis*. Thus, the prevalence of *Candida albicans* declined, reflecting the rising trend of non-*albicans Candida* species. This decline may be attributed to prophylactic antifungal use, which selectively eliminates *Candida albicans* due to its susceptibility while allowing resistant non-*albicans Candida* species to persist. This pattern is commonly observed in patients with hematological malignancies.

Among the non-*albicans Candida* species, *Candida tropicalis* emerged as the most dominant isolate in the current study, aligning with Li’s findings [12], whereas *Candida parapsilosis* was the most dominant species in the 2008–2012 study [14]. Multiple investigations have established that *Candida tropicalis* or *Candida parapsilosis* is the most frequently identified invasive non-*albicans Candida* species in various parts of mainland China [12, 14–16], whereas *Candida glabrata* was predominant in the southwestern region [17]. Furthermore, a study in a tertiary general hospital in Suining revealed that *Candida tropicalis* had the highest isolation rate among all *Candida* species [12].

Among patients with hematological malignancies, *Candida tropicalis* emerged as the predominant causative agent of candidemia, whereas *Candida albicans* was the leading cause in patients with other conditions [18]. Consistent with the current study, we also found that the detection rate of *Candida tropicalis* in the Hematology Ward was the highest (86.36%, 19/22). This could be attributed to the prevalent use of antifungal azoles in hematological patients, potentially contributing to an increase in non-*albicans Candida* infections and azole resistance [19]. These findings underscore the geographical variability in *Candida* species prevalence and emphasize the importance of conducting regional monitoring.

The rise of non-*albicans Candida* species is a cause for concern due to their potential for antifungal resistance [20]. Further analysis of drug susceptibility profiles revealed that triazole drugs (fluconazole, voriconazole, and itraconazole) exhibited poor sensitivity to *Candida tropicalis*, with sensitivity rates not exceeding 75.00%. Fortunately, resistance to fuconazole and voriconazole in *Candida tropicalis* was 25% and 16.67%, which is lower than the rates reported in Shandong Province [21], where resistance rates were 38.3% and 32.8%. Throughout the entire study period, no cases of resistance to amphotericin B were observed among any *Candida* species, aligning with findings from studies by Li [12], Arendrup MC [20], and Corentin Deckers [22].

Additionally, studies have demonstrated that the mortality rate of *Candida tropicalis* infections tends to be higher than that of other *Candida* infections [23]. This underscores the importance of clinicians in hematology departments considering the possibility of *Candida tropicalis* infections when empirically prescribing antifungal medications. Both China Hospital Invasive Fungal Surveillance Net (CHIF-NET) and multicenter monitoring data have revealed a significant upward trend in the resistance rate of *Candida tropicalis* to triazole [24, 25], along with a high degree of cross-resistance. Given this, echinocandins can be regarded as a frontline therapy for candidemia caused by *Candida tropicalis*. Echinocandin remains unaffected by resistance mechanisms that have evolved against azoles, owing to their distinct target within *Candida* species [26]. In a study conducted by Arikan-Akdagli [27] across 12 centers, no instances of echinocandin-resistant Candida were found. Consistent with these findings, occasional testing at our hospital has revealed that *Candida* species exhibit sensitivity to echinocandins. However, data on echinocandins in the current study are omitted due to the unconventional nature of the testing performed.

The mortality rate of patients with candidemia in the current study was 30.30% (40/132), which was close to the 31.34% reported by Guo Jing [28] and slightly lower than the 42–52% mortality rates reported by multiple international centers [29, 30]. *Candida* infections have a latent onset and lack characteristic clinical manifestations, making early diagnosis difficult for patients with candidemia [31]. Therefore, understanding their epidemiological characteristics and related risk factors that may affect outcomes is crucial for establishing timely and effective personalized diagnosis and treatment plans.

The results of the current study indicated that gastrointestinal surgery (non-tumor) was an independent risk factor for *Candida albicans* infection. The gastrointestinal tract is identified as a reservoir of *Candida albicans*,* Candida parapsilosis*, and *Candida tropicalis*, with *Candida albicans* (72.92%, 595/816) being the most frequently isolated species [32]. Gastrointestinal surgery can lead to the migration of *Candida albicans* from the gastrointestinal tract into the bloodstream, resulting in infection.

Factors closely related to the pathogenicity of *Candida* include impairment immune system impairment (due to diseases or immunosuppressive therapy), alterations in the normal flora (due to long-term use of broad-spectrum antibiotics), and disruptions to skin or mucosal barriers (caused by invasive medical procedures, burns, or wounds) [33]. Univariate analysis in the current study revealed that age, hypoproteinemia, septic shock, fever, respiratory diseases (non-tumor), central venous catheter, CRBSI/CLABSI, and ICU admissions were related to mortality. Multivariate logistic regression analysis revealed age and septic shock as risk factors for mortality in patients with candidemia.

In the current study, compared to the survival group, patients in the deceased group had a higher average age, consistent with previous research [34]. This may be attributed to declining physical and immune functions, a higher prevalence of comorbidities, and an increased risk of malignancy in older patients. Elderly individuals often have weakened immune function and are more likely to have chronic diseases, such as diabetes, cardiovascular or cerebrovascular diseases.

Septic shock, reflecting the severity of the infection, is a predominant factor leading to mortality, with hospital fatality rates ranging from 30 to 50%. *Candida* is a primary pathogen associated with septic shock, accounting for 23–38% of cases. When complicated by septic shock, candidemia becomes a severe condition, with mortality rates potentially surpassing 60% [35]. In the current study, non-survivors had a significantly higher incidence of septic shock than survivors (50.00% versus. 16.30%). Similarly, a study conducted by Liu et al. [36] found that septic shock (OR = 5.704, 95% CI: 2.639–12.326, *P* < 0.01) was an independent risk factor for mortality in patients with candidemia. These findings underscore the importance of timely therapy to improve the prognosis of elderly patients with septic shock.

Although our data were collected from two large hospitals, which can represent the Linyi region, several limitations of the current study should be noted. First, as a retrospective study restricted to the Linyi region, the findings should be interpreted with caution. Second, the patients enrolled in the current study did not present with certain comorbidities, such as intra-abdominal infections, intra-abdominal abscess, dialysis, endophthalmitis, or infective endocarditis, making the current study unsuitable for patients with these complications. Third, due to the lack of echinocandin testing in the ATB^®^ FUNGUS 3 assay, echinocandin susceptibility was not assessed. To address this limitation, future research must integrate echinocandin susceptibility testing into surveillance efforts. These issues will be addressed in future research.

In summary, although *Candida albicans* remains the dominant species, *Candida tropicalis* is increasing and exhibits poor sensitivity to triazole drugs, highlighting the necessity of antifungal susceptibility surveillance. Gastrointestinal surgery (non-tumor) was an independent risk factor for *Candida albicans* infection, emphasizing the importance of proper gastrointestinal surgery management. Moreover, age and septic shock were risk factors for mortality in patients with candidemia. Consequently, high-risk patients should receive effective antifungal therapy as soon as possible to control the infection and improve prognosis.

## Data Availability

The data set supporting the conclusions in this article is available from the corresponding author on reasonable request.

## Abbreviations

BSI: Bloodstream infections
ICU: Intensive care unit
AFST: Antifungal susceptibility test
MIC: Minimum inhibitory concentration
S: Susceptible
WT: Wild type
R: Resistant
OR: Odds ratio
CI: Confidence interval
CRBSI: Catheter-related bloodstream infections
CLABSI: Central line-associated bloodstream infections
CHIF-NET: China Hospital Invasive Fungal Surveillance Net
